# Aged green tea reduces high-fat diet-induced fat accumulation and inflammation via activating the AMP-activated protein kinase signaling pathway

**DOI:** 10.29219/fnr.v66.7923

**Published:** 2022-03-10

**Authors:** Ruohong Chen, Xingfei Lai, Limin Xiang, Qiuhua Li, Lingli Sun, Zhaoxiang Lai, Zhigang Li, Wenji Zhang, Shuai Wen, Junxi Cao, Shili Sun

**Affiliations:** Tea Research Institute, Guangdong Academy of Agricultural Sciences/Guangdong Provincial Key Laboratory of Tea Plant Resources Innovation and Utilization, Guangzhou, China

**Keywords:** anti-obesity, anti-inflammation, AMPK, aged green tea, metabolism

## Abstract

**Background:**

Obesity is a global public health concern and increases the risk of metabolic syndrome and other diseases. The anti-obesity effects of various plant-derived bioactive compounds, such as tea extracts, are well-established. The mechanisms underlying the anti-obesity activity of Jinxuan green tea (JXGT) from different storage years are still unclear.

**Objective:**

The aim of this study was to evaluate the effects of JXGTs from three different years on the high fat diet (HFD)-fed mouse model.

**Design:**

The mice were divided into six groups, the control group received normal diet and the obese model group received HFD. We analyzed the effects of JXGTs from 2005, 2008, and 2016 on HFD-fed obese mice over a period of 7 weeks.

**Results:**

The JXGTs reduced the body weight of the obese mice, and also alleviated fat accumulation and hepatic steatosis. Mechanistically, JXGTs increased the phosphorylation of AMP-activated protein kinase (p-AMPK)/AMP-activated protein kinase (AMPK) ratio, up-regulated carnitine acyl transferase 1A (CPT-1A), and down-regulated fatty acid synthase (FAS), Glycogen synthase kinase-3beta (GSK-3β), Peroxisome proliferator-activated receptor-gamma co-activator-1alpha (PGC-1α), Interleukin 6 (IL-6), and Tumour necrosis factor alpha (TNFα). Thus, JXGTs can alleviate HFD-induced obesity by inhibiting lipid biosynthesis and inflammation, thereby promoting fatty acid oxidation via the AMPK pathway.

**Discussion:**

The anti-obesity effect of three aged JXGTs were similar. However, JXGT2016 exhibited a more potent activation of AMPK, and JXGT2005 and JXGT2008 exhibited a more potent inhibiting glycogen synthase and inflammation effect. Furthermore, the polyphenol (–)-epicatechin (EC) showed the strongest positive correlation with the anti-obesity effect of JXGT.

**Conclusions:**

These findings demonstrate that JXGT treatment has a potential protection on HFD-induced obesity mice via activating the AMPK/CPT-1A and down-regulating FAS/GSK-3β/PGC-1α and IL-6/TNFα. Our study results also revealed that different storage time would not affect the anti-obesity and anti-inflammation effect of JXGT.

**Graphical abstract:**

**Figure:**
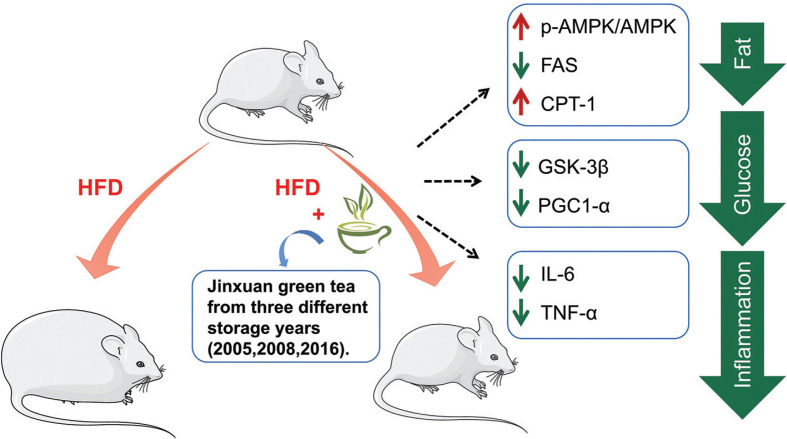

To access the supplementary material, please visit the article landing page

## Popular scientific summary

- This research study employed the water extracts of aged Jinxuan green tea on anti-obesity and anti-inflammation effects.
- The treatment of Jinxuan green tea significantly reduced body weight and fat accumulation in high-fat diet fed obese mice, and the different storage time would not affect the effect.
- The mechanism of Jinxuan green tea appears to be mediated by activating the AMPK/CPT-1A and down-regulating FAS/GSK-3β/PGC-1α and IL-6/TNFα.

Obesity results from the imbalance between high-energy intake and low-energy expenditure, and is currently a global health concern (1). It is a major risk factor of hypertension, type 2 diabetes, cancer, rheumatoid arthritis and cardiovascular diseases (2–5). Studies show that several plant-derived bioactive compounds can alleviate obesity without the side effects of conventional weight loss drugs (6–9).

Tea brewed from the fresh leaves of Camellia sinensis contains a variety of bioactive compounds including polysaccharides, polyphenols, and so on (10, 11). Several studies have demonstrated the anti-obesity effects of green tea, Fubrick tea, and black tea (12–14). The composition of the bioactive compounds in the different types of tea depends on the processing and fermentation. Green tea is a non-fermented tea, although its prolonged storage induces a slight natural fermentation that may alter its active components.

AMP-activated protein kinase (AMPK) controls lipid metabolism by modulating the CPT-1A and FAS pathways (15, 16). The AMPK activation also correlates with GSK-3β downregulation (17) and mitochondrial biogenesis *via* PGC-1α (18). In our previous studies, we found that different types of tea can alleviate obesity *via* AMPK activation. Furthermore, the weight-loss effect of green tea is associated with the AMPK/CPT-1A/FAS and GSK-3β/ PGC-1α pathways.

The aim of this study was to evaluate the effects of Jinxuan Green teas (JXGTs) from three different years on the high-fat diet (HFD)-fed mouse model for determining the effect of its prolonged storage on the anti-obesity components of green tea. We found that JXGTs alleviated HFD-induced weight gain by elevating the p-AMPK/AMPK ratio, and the activated AMPK mitigated lipid synthesis and balanced energy metabolism through the CPT-1A/FAS and GSK-3β/PGC-1α pathways, respectively. Furthermore, JXGTs inhibited obesity-induced inflammation by downregulating pro-inflammatory factors, such as IL-6 and TNFα. Taken together, JXGT mediates anti-obesity and anti-inflammatory effects that warrant further investigation.

## Materials and methods

### Preparation and characterization of lyophilized JXGT extract

Dried JXGT leaves from the years 2005, 2008, and 2016 were obtained from the Tea Research Institute, Guangdong Academy of Agricultural Sciences in China. As reported previously (19), the JXGT leaves were pulverized and extracted by boiling in water for 30 min (tea/water = 1:20 w/v). The tea extracts were concentrated by rotary evaporation to one-fifth of the original volume and dried by a vacuum freeze dryer. The content of free amino acids, total soluble sugar, polyphenols, caffeine, and catechin were measured by the ninhydrin method, anthrone-sulfuric acid colorimetric assay, Folin-phenol method, and high-performance liquid chromatography (HPLC), respectively, as previously reported (20–22).

### Establishment of obesity model in mice and treatment regimen

Male C57BL/6J mice (7 weeks old) were purchased from Beijing Huafukang Bioscience Co. Ltd. (Beijing, China) All experimental procedures were approved by the Ethics Committee of the institute, and performed according to the institutional guidelines for the care and use of laboratory animals. The protocols were approved by the Ethical Committee of Tea Research Institute. The mice were individually housed at 23 ± 2°C and 60 ± 15% humidity on a 12-h light/dark cycle, with free access to deionized water and basic feed. After a week of adaptation, the mice were randomly divided into the following six groups (*n* = 8 each): control (basic diet), model (HFD), positive control (HFD + 10 mg/kg/day atorvastatin), JXGT 2005 (HFD + 1000 mg/kg/day JXGT 2005), JXGT 2008 (HFD + 1000 mg/kg/day JXGT 2008), and JXGT 2016 (HFD + 1000 mg/kg/day JXGT 2016). The mice were given intragastric administration once a day for 7 weeks. The normal diet consisted of 18% proteins, 4% fats, 62% carbohydrates, 5% fiber, 8% minerals, and 3% vitamins for the control group. The calorific contribution of fats, proteins, and carbohydrates in the HFD were 45, 20, and 35% respectively for HFD-induced groups. Both feeds were prepared by the Guangdong Medical Laboratory Animal Center. Each group was provided with distilled water, and the body weight, food and water intake were recorded once a week.

### Tissue processing

After 7 weeks of treatment, the mice were anesthetized with 40 mg/kg pentobarbital following overnight fasting and euthanized by cervical dislocation. The whole blood was collected into heparinized tubes, and the sera were separated by centrifuging at 3,000 rpm for 10 min. The adipose tissues (including abdominal fat, intestinal fat, and perirenal fat) and liver were removed, washed with PBS, weighed, and frozen at –80°C for further analysis.

### Biochemical analysis

The serum levels of triglycerides (TGs), total cholesterol (TC), high-density lipoprotein cholesterol (HDL-C), and low-density lipoprotein cholesterol (LDL-C) were measured using commercially available kits (Nanjing Jiancheng Bioengineering Institute, China) according to the instructions.

### Protein extraction and Western blotting

Total protein was extracted from the liver using a protein extraction kit (Jiancheng Bioengineering Institute, Nanjing, China). Equal amounts of protein per sample were resolved by 10% SDS-PAGE and transferred to Polyvinylidene fluoride (PVDF) membranes. After blocking with 5% skimmed milk in Tris Buffered Saline with Tween-20 (TBST) for 1 h at room temperature, the proteins were incubated overnight with primary antibodies against AMPK (#2532S, Cell Signaling Technology, Danvers, MA, USA), p-AMPK (#2535S, CST), CPT-1A (15184-1-AP, Proteintech Group, Rosemont, USA), FAS (Abp51334, Abbkine, CA, USA), GSK-3β (#9315, CST), PGC-1α (2178S, CST), IL-6 (bs-0379R, Bioss, Beijing, China), TNFα (ab6671, Abcam, Cambridge, UK), and β-actin (Sigma-Aldrich, St Louis, MO, USA) at 4°C. The membranes were then probed with anti-rabbit secondary antibody IgG (HRP) (ab6721, Abcam) or anti-mouse secondary antibody IgG (HRP) (ab197767, Abcam) for 1 h at room temperature. After washing thrice with TBST, the blots were developed using a chemiluminescence reagent (P0018A, Shanghai Beyotime Biotechnology Co., Ltd, China), and the positive bands were visualized with a Gel Imaging System (General Electric, Fairfield, CT, USA). The band intensities were measured using the ImageJ software.

### Statistical analysis

All statistical analyses were performed using SPSS 16.0 (IBM, USA), and GraphPad Prism 7.0 (USA) was used to plot graphs. Multiple groups were compared by one-way analysis of variance (ANOVA) followed by Dunnett’s test. Independent Student’s t-test (two-tailed) was used for pairwise comparison. The correlation between factors was evaluated by Pearson correlation analysis. All data are presented as the means ± SD of at least three independent experiments, *P* < 0.05 was considered to be statistically significant.

## Results

### Prolonged storage affects the composition of JXGTs

As shown in Table 1, JXGT2005 and JXGT2008 had a higher water content compared with JXGT2016. Due to time-dependent degradation and oxygenation during storage, the content of free amino acids, soluble sugars, and tea polyphenols was significantly lower in the aged JXGT, as reported in our previous studies (23, 24).

**Table 1:** The components of Jinxuan Green teas from three different storage years

Constituent	JXGT2005	JXGT2008	JXGT2016
Free amino acid (%)	1.62 ± 0.03^a^	1.65 ± 0.06^a^	2.30 ± 0.04^b^
Soluble sugar (%)	6.61 ± 0.02^a^	6.25 ± 0.11^a^	7.80 ± 0.03^b^
Tea polyphenols (%)	30.77 ± 2.79^a^	30.54 ± 2.52^a^	32.65 ± 1.52^a^
GA	13.16 ± 1.23^a^	11.25 ± 1.88^a^	55.98 ± 3.04^b^
GC	5.87 ± 0.03^a^	5.86 ± 0.04^a^	9.04 ± 0.21^b^
EGC	1.11 ± 0.00^a^	1.11 ± 0.00^a^	1.06 ± 0.00^b^
C	1.54 ± 0.01^a^	1.64 ± 0.05^a^	2.31 ± 0.05^b^
CAFF	19.73 ± 0.29^a^	20.38 ± 0.14^a^	17.59 ± 0.32^b^
EC	3.53 ± 0.10^a^	4.15 ± 0.16^a^	4.72 ± 0.25^b^
EGCG	31.23 ± 0.41^a^	32.07 ± 0.13^a^	28.32 ± 0.26^b^
GCG	8.31 ± 0.14^a^	7.48 ± 0.20^b^	8.68 ± 0.30^a^
ECG	1.21 ± 0.02^a^	1.14 ± 0.04^a^	0.87 ± 0.04^a^
CG	5.89 ± 0.05^a^	5.87 ± 0.21^a^	5.88 ± 0.17^a^
Water (%)	9.50 ± 0.02^a^	9.20 ± 0.02^b^	4.64 ± 0.01^c^

The value is mean ± SD (*n* = 3). Values marked with different lower case letters in superscript format indicate significant difference, values marked with the same lower case letters in superscript format indicate no significant difference.

Note: (−)-epicatechin (EC), (−)-epigallocatechin (EGC), (−)-epicatechin gallate (ECG), (−)-epigallocatechin gallate (EGCG), (+)-catechin (C) and (+)-gallocatechin (GC), (−)-catechin gallate (CG) and (−)-gallocatechin gallate (GCG).

a,b,c The value of ingredients contents is mean ± SD (*n* = 3). Means followed by the same letter are not significantly different at *P* < 0.05.

### JXGTs reduced body weight in HFD-fed obese mice

**Fig. 1:**
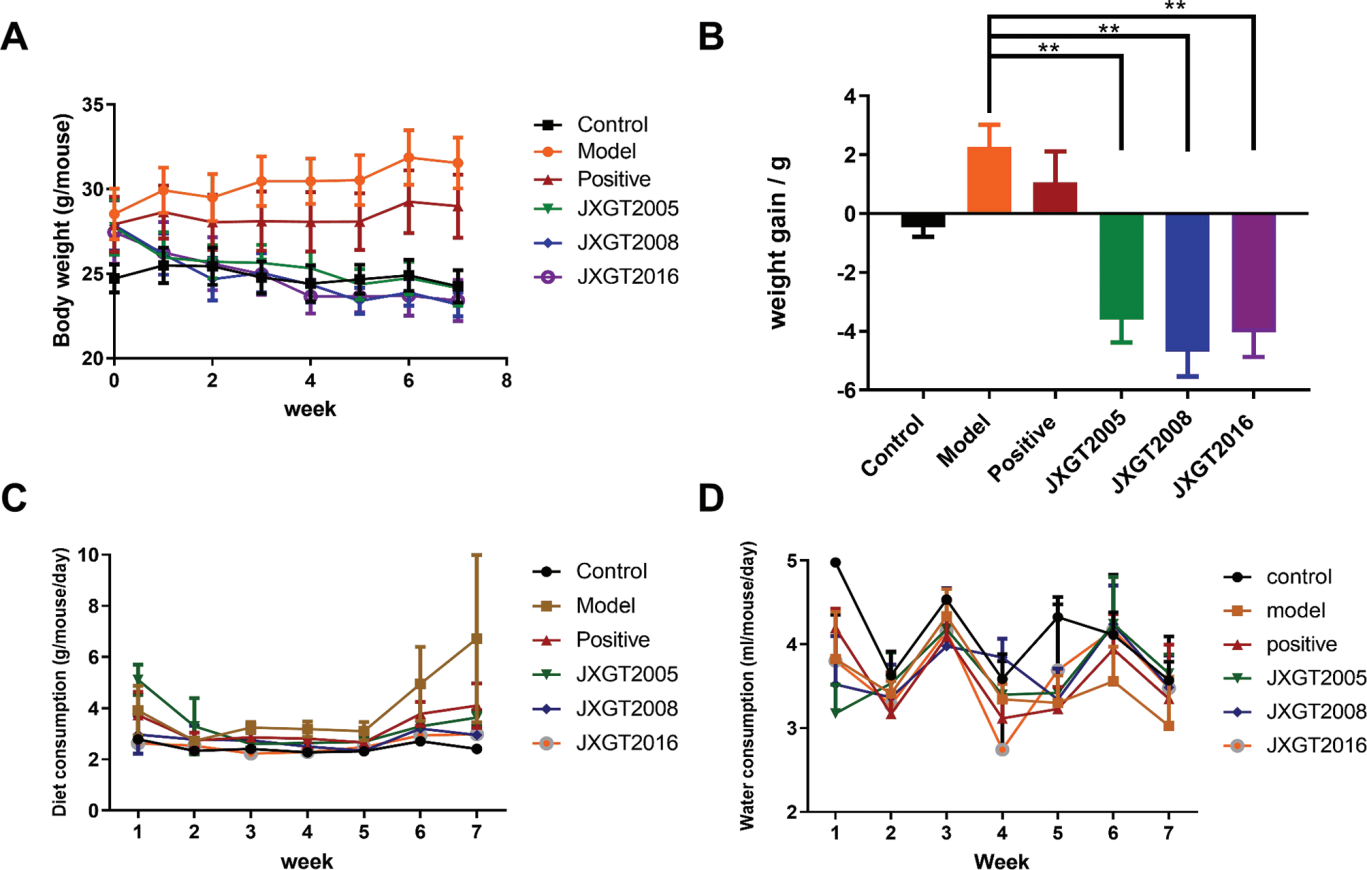
Effect of JXGTs on the body weight (A), weight gain (B), diet consumption (C), and water consumption (D) of HFD-fed obese mice. Data are presented as means ± SD (*n* = 8). **P < 0.01 and *P < 0.05.

As shown in Fig. 1A, HFD feeding for 7 weeks significantly increased the body weight of the mice compared with the normal diet-fed controls. In contrast, intragastric administration of JXGTs during the 7-week regimen significantly inhibited the HFD-induced weight gain (*P* < 0.01; Fig. 1B). The daily food and water intake did not show any marked differences among all groups (Fig. 1C and D). Taken together, JXGTs can prevent HFD-induced obesity without suppressing calorific intake.

### JXGTs attenuated fatty liver and adiposity in the HFD-fed obese mice

**Fig. 2:**
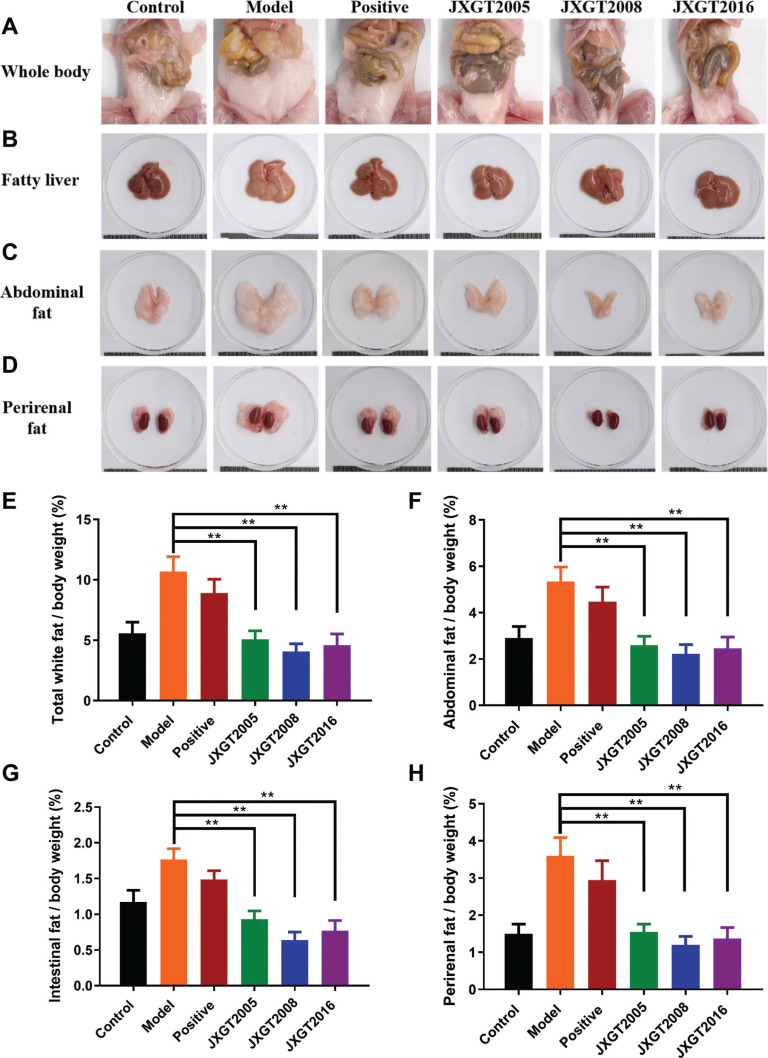
JXGTs attenuate fatty liver and adiposity in HFD-induced obese mice. Representative images of whole body (A), fatty liver (B), abdominal (C), and perirenal (D) white fat in all groups. The indices of total (E), abdominal (F), intestinal (G), and perirenal (H) white fat relative to body weight. Data are presented as the means ± SD (*n* = 8). **P < 0.01 and *P < 0.05.

The effects of JXGTs on fat accumulation were evaluated in terms of anatomical and biochemical indices. JXGTs markedly reduced the accumulation of white fat in the HFD-fed obese mice compared with the untreated mice (Fig. 2A). As shown in Fig. 2B, yellowish-brown fatty livers characterized by uneven surface were observed in mice fed with the HFD for 7 weeks compared with the healthy controls. JXGTs treatment protected the liver of the HFD-fed mice from steatosis. Furthermore, JXGTs also significantly decreased the size of the abdominal (Fig. 2C) and perirenal (Fig. 2D) fat tissue masses, especially in the JXGT2008 group. Consistent with this, JXGTs also reduced the total amount of white fat, and that of epididymal, intestinal and pararenal fat in the HFD-fed mice to near-baseline levels, and the effect was similar for the JXGTs from different storage years (*P* < 0.01; Fig. 2E–H). Thus, JXGT treatment can effectively attenuate HFD-induced fatty liver and adiposity. Furthermore, HFD markedly increased the serum levels of TGs, TC, high-density lipoprotein (HDL), and low-density lipoprotein (LDL). The supplementation of JXGT reversed the HFD-induced increment in TG (Supplementary Fig. 1A) but did not affect the other indices (Supplementary Fig. 1B–D).

### JXGTs activate AMPK-driven metabolic pathways

**Fig. 3:**
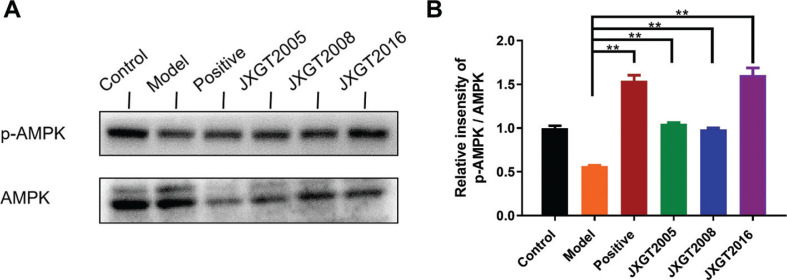
JXGTs activate AMPK phosphorylation. (A) Immunoblot showing AMPK protein levels in the liver of differentially treated mice and (B) densitometric quantification. Data are presented as means ± SD (*n* = 3). **P < 0.01 and *P < 0.05.

**Fig. 4:**
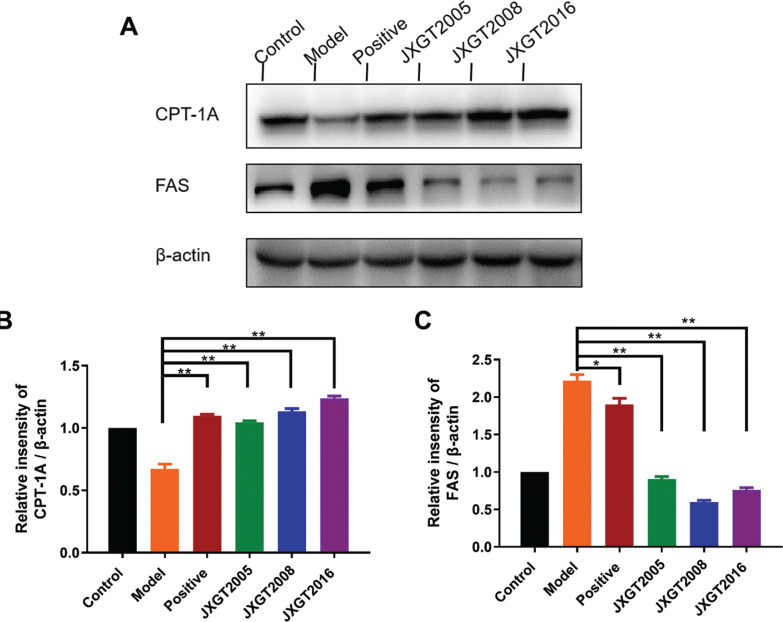
JXGTs upregulate CPT-1 and inhibit FAS expression. (A) Immunoblot showing expression levels of CPT-1 and FAS protein in mouse liver and densitometric quantification of (B) CPT-1 and (C) FAS. Data are presented as the means ± SD (*n* = 3). **P < 0.01, and *P < 0.05.

**Fig. 5:**
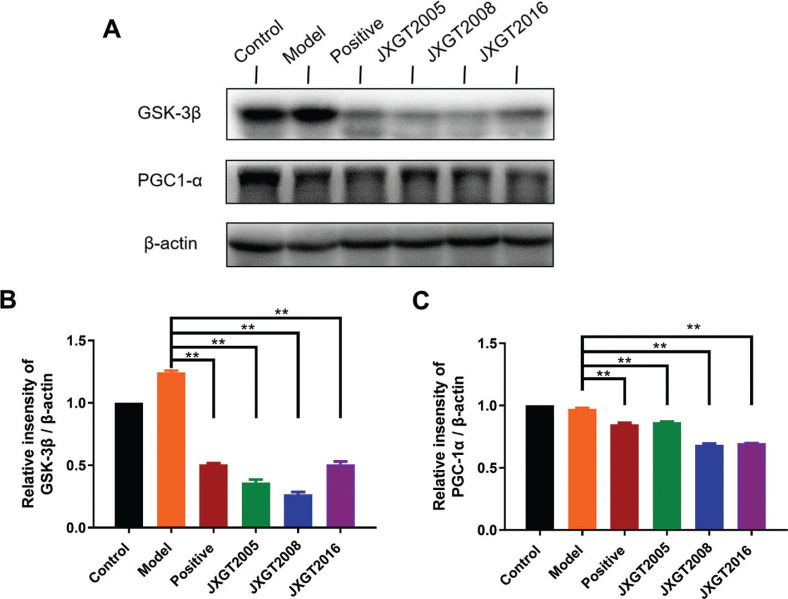
JXGTs upregulate PGC-1α and inhibit GSK-3β expression. (A) Immunoblot showing expression levels of GSK-3β and PGC-1α protein in mouse liver and densitometric quantification of (B) GSK-3β and (C) PGC-1α. Data are presented as means ± SD (*n* = 3). **P < 0.01 and *P < 0.05.

AMPK plays an important role in energy metabolism by stimulating fatty acid oxidation. The HFD-fed obese mice had significantly a lower level of p-AMPK in the liver, which was reversed by JXGT treatment (Fig. 3A). Consistent with this, HFD decreased the p-AMPK/AMPK ratio by 44% compared with that in healthy controls, and was restored by JXGTs from the different storage years (Fig. 3B). CPT-1 is the rate-limiting enzyme of fatty acid oxidation, and FAS is a key enzyme involved in fatty acid synthesis. As shown in Fig. 4A, CPT-1 and FAS were, respectively, downregulated and upregulated in the liver of obese mice, and their expression levels were significantly reversed by JXGT treatment (Fig. 4B). GSK-3β and PGC1-α are the key protein kinases involved in energy metabolism. As shown in Fig. 5A, GSK-3β was up-regulated in the HFD-fed mice and decreased by JXGT treatment. In addition, PGC1-α was down-regulated in the JXGT-treated groups (Fig. 5B and C).

### JXGTs inhibit IL-6 and TNF-α expression

**Fig. 6:**
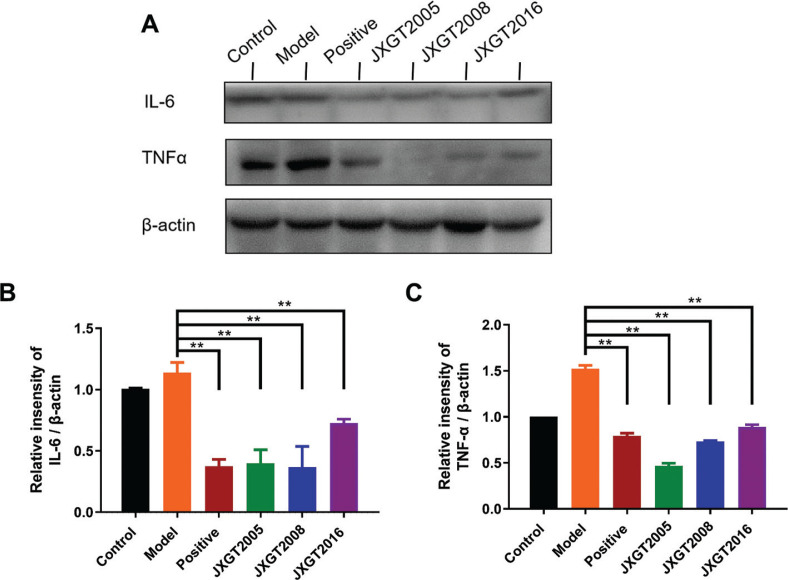
JXGTs inhibit IL-6 and TNF-α expression. (A) Immunoblot showing IL-6 and TNF-α protein levels in mouse liver and densitometric quantification of (B) IL-6 and (C) TNF-α. Data are presented as means ± SD (*n* = 3). **P < 0.01 and *P < 0.05.

Obesity is usually associated with an increase in inflammation, and high in situ levels of IL-6 and TNFα, which can aggravate liver injury and weaken the hepatic glucolipid and lipid metabolism (25). As shown in Fig. 6, IL-6 and TNFα levels were significantly higher in the liver tissues of obese mice. Treatment with the different JXGTs significantly decreased the levels of both factors compared with that in the untreated obese mice (*P* < 0.01).

### Correlation analysis

**Fig. 7:**
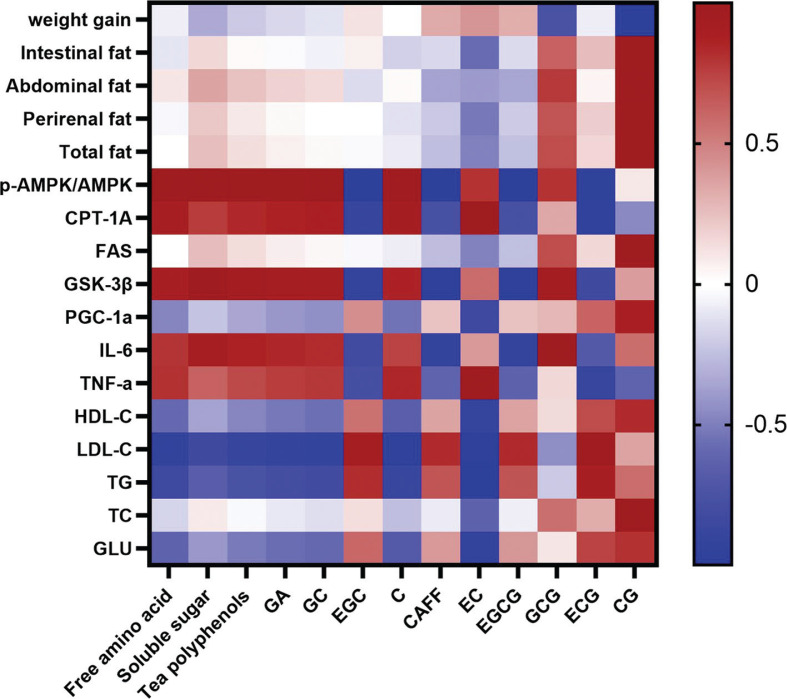
The correlation between the phytochemicals and obesity parameters and inflammation indices in the different experimental groups. (−)-epicatechin (EC), (−)-epigallocatechin (EGC), (−)-epicatechin gallate (ECG), (−)-epigallocatechin gallate (EGCG), (+)-catechin (C) and (+)-gallocatechin (GC), (−)-catechin gallate (CG) and (−)-gallocatechin gallate (GCG). Significant correlations are annotated by **P < 0.01 and *P < 0.05.

To further evaluate the role of tea-derived phytochemicals against obesity, we analyzed the Pearson correlation between the phytochemical composition of JXGTs and various parameters of obesity and inflammation (Fig. 7). The content of tea polyphenols, amino acids and soluble sugar was positively correlated with AMPK pathway activation, as well as most anti-inflammatory parameters. Furthermore, GCG (GCG) and catechin gallate (CG) were positively correlated with the loss of body weight.

## Discussion

Obesity is primarily a result of increased consumption of sugars and fats, and lack of physical exercise, along with aberrant fatty acid biosynthesis and degradation (26). It is a major health problem worldwide (27), and is accompanied by several hormonal and inflammatory disturbances that increase the risk of diabetes (28), hypertension (3), dyslipidemia and metabolic syndrome (29). The commonly prescribed weight-loss drugs like orlistat, sibutramine, and rimonabant cause side effects, such as oily stools and flatulence (30). Several studies have identified plant-derived bioactive compounds with significant anti-obesity and weight-loss effects (31–34). For instance, tea brewed from fresh leaves of *C. sinensis* has several beneficial effects. Depending on the extent and method of fermentation, tea is classified into the non-fermented green tea, lightly fermented yellow tea and white tea, partially fermented oolong tea, completely fermented black tea, and post-fermented dark tea (35). Green tea, in particular, has exhibited protective effects against skin photoaging, stress, neurodegeneration, hypertrophy, hypolipidemia, inflammation, and obesity (36–39).

Atorvastatin is one of the most widely prescribed drugs and the most widely prescribed statin in the world (40), which is widely used as a positive control to lower elevated lipid levels and anti-obesity by difference dosages (1–80 mg/kg/day) treatment in the HFD induced model (19, 41, 42). Therefore, positive control group is treated with a relatively low-dose atorvastatin (10 mg/kg/day) in this study. In this study, we compared the potential anti-obesity effects of JXGTs from different storage years on HFD-fed mice. Although the JXGTs had little effect on the calorific intake of the mice, they significantly reduced body weight and fat accumulation at multiple anatomical sites, and the duration of storage had no significant effect on the anti-obesity effects of JXGT (23).

The liver is the central organ of lipid storage and metabolism. The consumption of high amount of dietary fat leads to liver steatosis (43). AMPK is the main sensor of energy status in eukaryotic cells, and thus, coordinates the growth and metabolism of specific tissues. The AMPK/p-AMPK is highly sensitive to energetic stress, and the liver-specific AMPK activation reprograms lipid metabolism and mitigates diet-induced obesity in mice (44). Studies show that the green tea extract and specific bioactive compounds like maslinic acid and EGCG can reduce obesity in mouse and zebrafish obesity models, respectively, through AMPK activation (45–47). In our previous studies, we found that aged oolong tea and Hakka stir-fried tea protected mice against obesity by activating the AMPK signaling pathway (19, 23, 24). As the JXGTs also markedly induced AMPK phosphorylation and the p-AMPK/AMPK ratio in liver tissues, their anti-obesity effects are also likely mediated via the AMPK signaling pathway.

The limiting factor of lipogenesis is malonyl-CoA, which is also an important precursor of the lipid biosynthetic pathway. AMPK activation decreases cellular malonyl-CoA levels, which, in turn, upregulates CPT1 (48). And FAS is a major regulator of lipogenic protein, and its activity is also regulated by AMPK. Then we demonstrated that aged JXGT significantly increased the expression of CPT1A (Fig. 4A and B) and inhibited the protein level of FAS (Fig. 4A and C). Our data established that the activation of AMPK/CPT-1A pathway and the inhibition of FAS pathways might be potential targets for JXGT treatment to prevent hepatic lipid accumulation.

The activation of AMPK not only inhibits the lipid synthesis and increases lipid oxidation but also the glucose synthesis in liver (49). GSK3β is a key enzyme of glycogen synthesis, and is elevated in both human subjects and animal models with diabetes. AMPK activation is associated with inhibition of GSK3β (50), and JXGT-mediated activation of AMPK in our HFD-fed model also decreased the obesity-induced overexpression of GSK3β. Another downstream target of AMPK is the transcription factor PGC-1α, which increases the expression of genes involved in mitochondrial biogenesis (51). Studies show that the activation of AMPK can up-regulate PGC-1α and ultimately promote mitochondrial biogenesis (52, 53). In this study, the aged JXGTs decreased the levels of IL-6 and TNFα, which is indicative of their potent anti-inflammatory effect. The effects of these three JXGTs on body weight were similar (Figs. 1 and 2). However, the ratio of p-AMPK/AMPK in the JXGT2016 group was higher than that in the JXGT2005 and JXGT2008 groups (Fig. 3). And JXGT2005 and JXGT2008 groups decreased the levels of GSK-3β (Fig. 5B), IL-6, and TNFα (Fig. 6) much lower than the JXGT2016 group, which is indicative of their potent inhibited glycogen synthesis and anti-inflammatory effect. Our study results showed that different storage years of JXGT can significantly attenuate body weight gain by HFD through its increased lipid metabolism, inhibited glycogen synthesis, and anti-inflammatory functions related to p-AMPK activation.

The major bioactive component in green tea are the polyphenol compounds that constitute 24−36% of the dry weight, followed by protein (15%), lignin (7%), amino acids (3−4%), caffeine (2−4%), organic acids (2%) and chlorophyll (0.5%) (54). Most of the beneficial effects of green tea are attributed to the high polyphenol content (55). We found that the content of free amino acids, soluble sugar, and tea polyphenols was positively correlated with p-AMPK levels and negatively correlated with the serum levels of TG, HDL-C, and LDL-C, whereas no significant correlation was observed with fat accumulation (Fig. 7). The major polyphenols of green tea include EC, (−)-epigallocatechin (EGC), (−)-epicatechin 3-gallate (ECG), (−)-epigallocatechin 3-gallate (EGCG), (+)-catechin (C), and (+)-gallocatechin (GC), along with smaller amounts of (−)-catechin gallate (CG) and (−)-c (GCG) (12). Daily consumption of green tea extracts, especially EGCG, has been shown to increase fat oxidation and energy expenditure (55, 56). In addition, CG, EGC, ECG, and EGCG can suppress intracellular lipid accumulation in 3T3-L1 cells (57). Gallic acid (GA) inhibits lipid accumulation via the activation of AMPK in HepG2 cells (58). In this study, we analyzed the levels of specific polyphenols in the JXGTs by HPLC-MS (Table 1), and then revealed that GA, GC, C, EC and GCG were positively correlated with the AMPK pathway (Fig. 7). The correlation between the phytochemicals and obesity and inflammation indices in the different treatment groups was in agreement with the previous study. Whereas EGC, EGCG, ECG, and caffeine (CAFF) had a negative correlation, EC showed the strongest correlation with weight gain, and GCG and CG were positively correlated with fat accumulation (Fig. 7). Our data revealed that EGCG, as a portion of phytochemicals of JXGT, might have a opposite dose-dependent effect of AMPK activation in our used dose range. However, it does not mean that EGCG inhibits the activation of AMPK. However, the exact anti-obesity and anti-inflammatory effects of the different polyphenols need to be explored further. Furthermore, the possible synergistic effects of the different bioactive compounds of JXGTs need to be investigated. For instance, the consumption of caffeine and EGCG synergistically increased fat oxidation and energy expenditure (59).

## Conclusion

JXGTs reduced white fat accumulation, increased lipid metabolism, and inhibited glycogen synthesis in the HFD-fed obese mice by targeting FAS, GSK-3β, and the AMPK/CPT1A pathway. In addition, JXGT reduced inflammation by downregulating IL-6 and TNFα. The storage duration had no significant effect on the activity of JXGT. Finally, the polyphenol EC showed a significant positive correlation with AMPK activation and weight gain Taken together, JXGT is a promising therapeutic agent against obesity and metabolic disorders, and different storage time would not affect the anti-obesity and anti-inflammation effects of JXGT.

## Conflicts of interest and funding

The authors declare no conflict of interest. This study was funded by the “14th Five-Year Plan” team-building projects of Guangdong Academy of Agricultural Sciences [Grant Nos. 202126TD]; Guangdong Basic and Applied Basic Research Foundation [Grant Nos. 2020A1515011266, 2021A1515010958]; Guangzhou Science and Technology Plan Projects [Grant Nos. 202102020047, 202002030202]; Key-Area Research and Development Program of Guangdong Province [Grant Nos. 2020B0202080003]; Maoming Science and Technology Program (Grant Nos. mmkj2020045); Zhanjiang Science and Technology Program (Grant Nos. 2020A03014); Innovation Fund projects of Guangdong Academy of Agricultural Sciences (Grant Nos. 202115, 202035); Special fund for scientific innovation strategy-construction of high level Academy of Agriculture Science (Grant Nos. R2019PY-JX004); the Innovation Fund projects of Guangdong Key Laboratory of Tea Plant Resources Innovation and Utilization (Grant Nos. 2021CX02). Funders did not have any role in study design, data collection, and data analysis.

## Authorship contributions

All authors contributed to the design and conduct of the study, data collection and analysis, data interpretation, and manuscript writing.

## Supplementary Material

Aged green tea reduces high-fat diet-induced fat accumulation and inflammation via activating the AMP-activated protein kinase signaling pathway
