# Supplementary material for: Aged green tea reduces high-fat diet-induced fat accumulation and inflammation via activating the AMP-activated protein kinase signaling pathway
Source: Food Nutr Res. 2022 Mar 10;66:10.29219/fnr.v66.7923. doi: 10.29219/fnr.v66.7923 (PMC8941417; doi:10.29219/fnr.v66.7923)
Supplement: Aged green tea reduces high-fat diet-induced fat accumulation and inflammation via activating the AMP-activated protein kinase signaling pathway [file FNR-66-7923-s001.docx]

**Fig S1. Serum levels of triglycerides (TG), total cholesterol (TC), high-density lipoprotein (HDL), and low-density lipoprotein (LDL).**


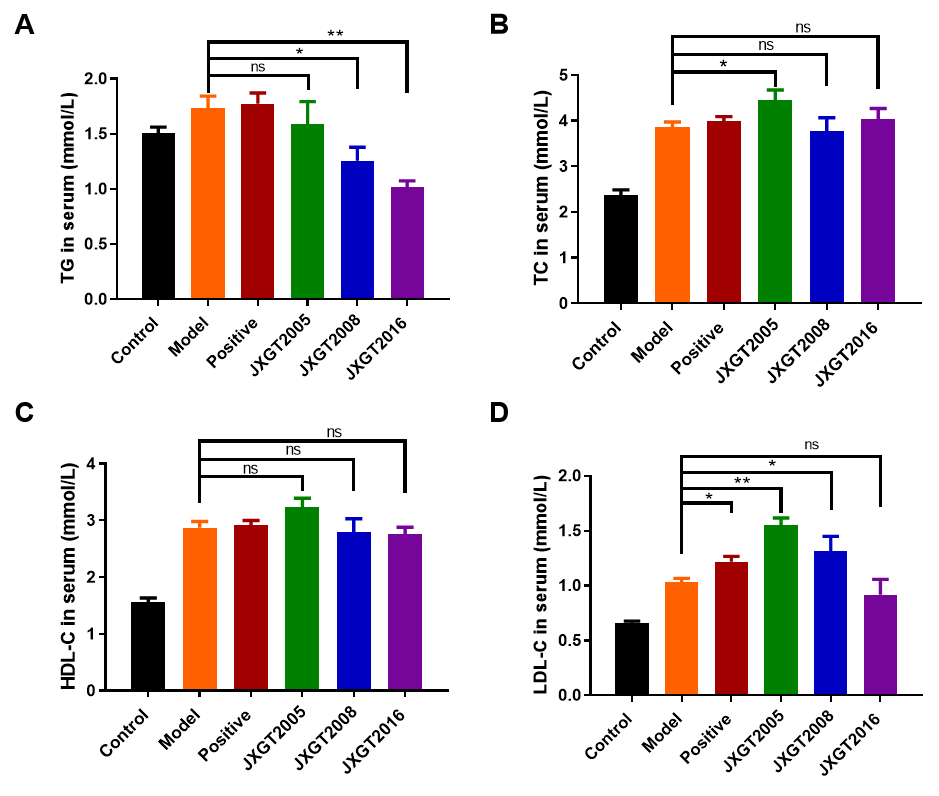

Data are presented as the means ± SD (n=8). **P < 0.01, and *P < 0.05.
